# Multimodal Imaging of the Fundus

**DOI:** 10.1155/2013/956761

**Published:** 2013-06-18

**Authors:** Atsushi Hayashi, Osman Cekic, Masanori Hangai, Yoshinori Mitamura, Andreas W. A. Weinberger

**Affiliations:** ^1^Department of Ophthalmology, Graduate School of Medicine and Pharmaceutical Sciences, University of Toyama, Toyama 930-0194, Japan; ^2^Department of Ophthalmology, Marmara University School of Medicine, 34903 Istanbul, Turkey; ^3^Department of Ophthalmology, Kurume University School of Medicine, Fukuoka 830-0011, Japan; ^4^Department of Ophthalmology, Institute of Health Biosciences, University of Tokushima Graduate School, Tokushima 770-8503, Japan; ^5^Department of Ophthalmology, RWTH Aachen University, 52074 Aachen, Germany

Retinal imaging devices made dramatic progress in these several years. These new devices reveal various anatomical and functional changes in the fundus by high resolution images, which may develop and improve diagnosis, treatment, and surgical maneuver.

The special issue on multimodal imaging of the fundus published one review article and five clinical research articles. The review article of Y. Mitamura et al. showed that the three lines in the outer retina detected by spectral-domain optical coherence tomography (SD-OCT) could serve as hallmarks for evaluation of photoreceptor condition in retinitis pigmentosa and recovery process after macular hole surgery. Of the five clinical research articles, N. F. Mokwa et al. compared sensitivity and specificity of imaging techniques of color fundus photography, fluorescein angiography, and SD-OCT for detecting age-related macular degeneration (AMD) and activity of choroidal neovascularization (CNV). F. Pichi et al. evaluated multimodal visualization of retinal genetic diseases by fundus autofluorescence, FA, indocyanine green (ICG) angiography, and SD-OCT to monitor progression of the diseases. O. A. Osmanbasoglu et al. analyzed diurnal variation of central choroidal thickness by enhanced depth imaging technique of SD-OCT in healthy emmetropic subjects. M. Şahin et al. reported hyperautofluorescence after cataract surgery, and J. M. Muñoz et al. compared ranges of contrast values in autofluorescence imaging between 2 fundus cameras. 

All the six articles in this special issue underwent a rigorous peer-review process.

